# Proteomics approaches: A review regarding an importance of proteome analyses in understanding the pathogens and diseases

**DOI:** 10.3389/fvets.2022.1079359

**Published:** 2022-12-15

**Authors:** Muhammad Zubair, Jia Wang, Yanfei Yu, Muhammad Faisal, Mingpu Qi, Abid Ullah Shah, Zhixin Feng, Guoqing Shao, Yu Wang, Qiyan Xiong

**Affiliations:** ^1^Key Laboratory of Veterinary Biological Engineering and Technology, Ministry of Agriculture, Institute of Veterinary Medicine, Jiangsu Academy of Agricultural Sciences, Nanjing, China; ^2^School of Food and Biological Engineering, Jiangsu University, Zhenjiang, China; ^3^College of Veterinary Medicine, Nanjing Agricultural University, Nanjing, China; ^4^Division of Hematology, Department of Medicine, The Ohio State University College of Medicine, The Ohio State University Comprehensive Cancer Center, Columbus, OH, United States; ^5^College of Veterinary Medicine, Huazhong Agricultural University, Wuhan, China; ^6^National Research Centre of Engineering and Technology for Veterinary Biologicals, Institute of Veterinary Immunology and Engineering, Jiangsu Academy of Agricultural Sciences, Nanjing, China; ^7^China Pharmaceutical University, Nanjing, China; ^8^School of Life Sciences, Jiangsu University, Zhenjiang, China

**Keywords:** proteomics, secretome, diseases, pathogenesis, diagnostics

## Abstract

Proteomics is playing an increasingly important role in identifying pathogens, emerging and re-emerging infectious agents, understanding pathogenesis, and diagnosis of diseases. Recently, more advanced and sophisticated proteomics technologies have transformed disease diagnostics and vaccines development. The detection of pathogens is made possible by more accurate and time-constrained technologies, resulting in an early diagnosis. More detailed and comprehensive information regarding the proteome of any noxious agent is made possible by combining mass spectrometry with various gel-based or short-gun proteomics approaches recently. MALDI-ToF has been proved quite useful in identifying and distinguishing bacterial pathogens. Other quantitative approaches are doing their best to investigate bacterial virulent factors, diagnostic markers and vaccine candidates. Proteomics is also helping in the identification of secreted proteins and their virulence-related functions. This review aims to highlight the role of cutting-edge proteomics approaches in better understanding the functional genomics of pathogens. This also underlines the limitations of proteomics in bacterial secretome research.

## 1. Introduction

**Figure 1:**
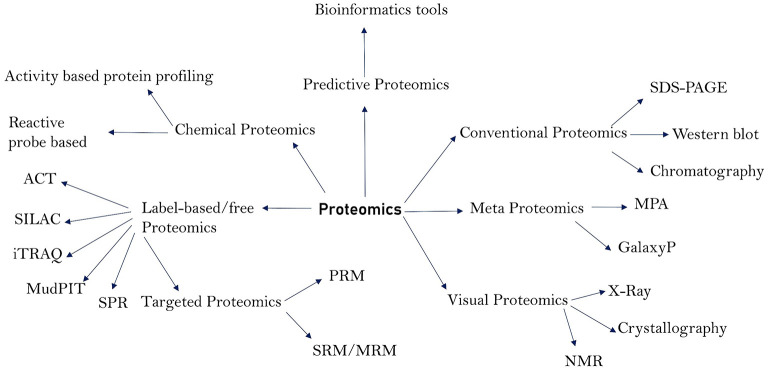
Major proteomics techniques and their subdivisions.

The total protein content of an organism is referred to as the proteome. The proteome, particularly of prokaryotic cells, has a wide range of roles and pathogenic properties, and proteomics is the study of these functions and characteristics (1). Proteomics has contributed not only to the discovery of pathogen virulence components, but also to the research of pathogen structural makeup, pathogenesis, disease diagnosis, and vaccine development or design (2–4). Proteins from bacteria and viruses act as virulent agents in the transmission of diseases in humans and animals. Membrane proteins (5), cell surface proteins, and secreted proteins are among the most important, as they play a crucial role in pathogenicity and have been extensively researched utilizing proteomics techniques (6–8). These proteins function as enzymes, transport molecules, toxins, adhesins, invasive, evasive, and receptors, and hence play a crucial role in the initiation and course of disease. Proteomics methods have vastly improved in the recent decade, making it possible to search for these critical proteins and examine their structures, molecular functions, and role in disease. Proteomics has been useful in identifying the microorganisms that cause various diseases and their architecture. Because genomics can only provide information on the pathophysiology of a disease, it is unable to expound on the cell state and pathogenic actions of the molecules that cause illness onset. Proteins are well-recognized for depicting the state of a disease by informing the pathogenic components that are the foundations for illness initiation (9). As a result, understanding the functions of such proteins is critical for understanding the pathophysiology, diagnosis, control, and therapy of infectious illnesses. Many proteomics technologies have been created over time and have shown to be invaluable in the study of pathogens and course of illnesses. Traditional proteomics techniques such as chromatography and western blotting have been utilized for a long time. Gel-based techniques such as 1-DE (1-Dimensional Gel Electrophoresis), 2-DE (2-Dimensional Gel Electrophoresis), and 2-DDGE (2-dimensional Differential Gel Electrophoresis) assisted in protein separation and identification (10). Low abundant proteins in the sample can be fractioned by using isoelectric fractionators followed by 2-D gels. Depending on the isoelectric focusing (IEF), low abundant proteins are concentrated making identification and quantification more reliable. Some commonly used fractionators are Rotofor (BioRad) and Zoom IEF fractionator (Invitrogen) (11). The combination of Gel Electrophoresis with Mass Spectrometry (2-DE-MS) improved the accuracy of protein identification. Isotope-Coded Affinity Tag (ICAT), Stable Isotopic Labeling with Amino Acids (SILAC), and Isobaric tag for relative and absolute quantification (iTRAQ) are some of the new quantitative approaches that have emerged as a result of advances in proteomics (1, 12). These modern quantitative approaches include surface plasmon resonance (SPR) for protein-protein interaction and Multidimensional protein identification technology (MudPIT), both of which are label-free tools commonly utilized for protein identification (13, 14). Absolute quantification of proteins can be accomplished using a variety of strategies, including absolute quantification using protein epitope signature tags (PrEST), protein standard absolute quantification (PSAQ), and intensity-based absolute quantification (iBAQ) (15–17). Membrane coated nanosponges paired with quantitative proteomics methods have recently been discovered to be a powerful source for identifying virulence factors (18). Types of proteomics techniques and their sub-divisions are depicted in Figure 1. Our understanding of infectious diseases, causative agents, and their diagnosis has increased over time due to advances in proteomics. As a result, the goal of this study was to shed light on the function of various proteomics methods in elucidating the pathophysiology, diagnosis, and causative agents of infectious diseases that affect humans and animals. In addition, we discussed the limitations of proteomics in terms of identifying pathogenic secreted proteins, as well as its future prospects.

## 2. Role of proteomics in identifying pathogens

The first stage in diagnosing a disease is to identify the causal agent, since their precise and detailed identification and confirmation aids in the prevention of illness transmission and knowledge of its epidemiology (2). Biochemical features, Gram staining, and carbohydrate metabolism are some of the traditional methods for identifying bacteria that have been used for a long time. Proteomics technologies, such as Mass Spectrometry (MS), have recently become popular for precisely identifying and confirming bacterial infections (19, 20). Proteomics methods are commonly used to identify pathogen structure and other components that contribute to virulence. Proteomics methods are being used to describe the structures of both bacterial and viral pathogens, with the goal of not only identifying structural and non-structural proteins involved in virulence, but also investigating metabolic and physiological factors. Classification of un-sequenced microorganisms has been made easier by using capLC-MS/MS on an Orbitrap (21).

Proteomics has been used to identify bacterial infections that cause various disorders. The use of proteomics methods to identify bacterial communities in surface and soil samples has also been done. Samples were gathered from children's books in Texas and California libraries, and the Orbitrap FusionTM TribridTM mass spectrometer identified a variety of non-pathogenic and harmful bacteria species. *S. haemolyticus, S. pneumoniae*, and *A. baumannii* were the most commonly discovered pathogenic species, causing skin infections and Multidrug-Resistant Tuberculosis (MDR) correspondingly (22). *Streptomyces violaceoruber, Streptomyces albus*, and *Streptomyces badius* were identified using MALDI-ToF-MS from soil samples collected in Algeria's Sahara (23). Mass spectrometry's most important and revolutionary role is in clinical microbiology, where it has shown to be a useful tool for rapidly identifying infectious pathogens at species level. Traditional methods for identifying a pathogen take longer, resulting in a serious illness condition before it can be treated (24). Antibiotic resistance develops as a result of the use of broad-spectrum antibiotics prior to the identification of the causative agent, as well as a detrimental influence on the patient's health (25). Forensic proteomics is another growing tool to identify bacterial species in a given sample. This method is based on identification of unique peptides and facing few challenges i.e., signature erosion (loss of signature sequences due to the addition of new sequences of identified species in database), absence of statistical precision and limited database (26).

Body fluids such as urine, milk, and blood are the most acceptable samples for microbe identification, and proteomics has done an excellent job of identifying microbes from these samples (27–29), as well as others such as cerebrospinal fluid, joint cavity fluid, vitreous fluid, and pleural fluid (27, 30).

### 2.1. Identification of pathogens from urine samples

By creating a specific reference urine database called Urinf, 90% of 500 samples were accurately diagnosed using MALDI-ToF (31). MALDI-ToF-MS was used to successfully identify *Corynebacterium rigelii*, a pathogen that causes urinary tract infections, from a case of urosepsis in a 67-year-old female patient (32). The urine-short incubation MALDI-TOF (U-si-MALDI-ToF) method was created mainly for the detection of *E. coli*, a bacteria that causes urinary tract infections. Using this technology, 86% of Gram-negative bacteria responsible for urinary tract infections, such as *E. coli, Klebsiella pneumoniae*, and Enterobacteriaceae, were discovered (33). Mass spectrometry has recently been combined with other technologies to improve identification accuracy. The Alfred 60 method was used in conjunction with MALDI-ToF-MS to detect bacteria that cause urinary tract infections. For most positive samples, combined technique proved more reliable and accurate in identifying uropathogens (25). Combining mass spectrometry with other screening technologies like flow cytometry saves time while improving identification quality (34). Urine samples were initially screened using a Sysmex (UF-1000i) flow 36 cytometer before being sent to the MALDI-ToF-MS. This method correctly detected 86.1% Gram-negative bacteria without any microorganism misidentification (35). Combining flow cytometry, such as the UF-5000i, with mass spectrometry reduces the time it takes to identify etiological agents responsible for urinary tract infections from 24 to 1 h (36). When MALDI-ToF-MS was paired with Urine Analysis (93.4 and 96.3%), sensitivity and specificity for the detection of urinary pathogens from urine samples were enhanced and more reliable than when MALDI-ToF-MS was used alone (86.6 and 91.5%) (37). Leptospires that cause leptospirosis were discovered using mass spectrometry and whole cell protein spectra. MALDI-ToF-MS also identified whole cells of leptospires with spikes in urine samples (38). Because of its greater sensitivity and specificity, researchers prefer LC-MS-MS to MALDI-ToF-MS. In light of this, a method for identifying urinary tract pathogens utilizing specific LC-MS-MS peptide signatures was devised. This targeted proteomics technique identified urinary tract infections in 97% of patients without the need for a culture and in < 4 h, proving to be the most rapid and reliable method for pathogen identification in urinary tract infections (39). Although it is clear that the proteomics tool of mass spectrometry has evolved as a viable approach for identifying urinary tract pathogens, the culture-independent MALDI-ToF approach can only identify pathogens in single microbial urine samples (40).

### 2.2. Identification of pathogens from blood samples

In the same way that mass spectrometry has made it easier and faster to identify pathogens in blood, its combination with other technologies has made it considerably more effective in urine analysis. The bacteria in blood culture samples were identified using a comparative analysis. In comparison to the SepsiTyper kit approach, which identified 99% (184/186) isolates, MALDI-TOF-MS was able to identify 90% (168/186) of them. As a result, it was determined that MALDI-ToF-MS analysis is recommended for bacterial identification in blood cultures due to its speed and ease of use (41). MALDI-ToF-MS identified 93.43% (185/198) Gram-negative germs and 78.43% (275/350) Gram-positive bacteria from blood cultures, with specificity and sensitivity of 84.7 and 77.5%, respectively, in another investigation (42). Positive blood cultures were quickly cultured on solid media before being identified using MALD-ToF-MS, which proved to be a reliable method for bacterial identification. At 3, 5, and 24 h, this approach correctly identified bacteria at the species level with 64.1, 85.0, and 94.1%, respectively. This is thought to be a viable method for identifying bacteria directly (43). Bacteria were enhanced in a blood sample using magnetite (Fe_3_O_4_) magnetic beads modified with human IgG (IgG@Fe3O4) and MALDI-ToF-MS, which showed to be a more sensitive method with less time than other culture-based methods. Bacteria with a concentration of 105 CFU/100 μl whole blood sample were identified quickly (44). MALDI-ToF-MS was used to analyze spiked blood culture samples, and the efficiency was found to be comparable to SepsiTyper (94.4%). This approach identified 82% Gram-positive bacteria in blood samples and was more sensitive (92.8%) for Gram-negative bacteria (45). The combination of MALDI-ToF-MS with immune-affinity has yielded highly consistent findings for bacterial identification at low concentrations (500 cells/ml for blood serum and 8,000 cells/ml for whole blood samples). Within roughly 4 h, this combination technique was able to identify *S. aureus* and *E. coli* in clinical samples (29).

### 2.3. Identification of pathogens from milk samples

Another essential body fluid for detecting germs that cause diseases in humans and animals is milk. The only reliable source for identifying bacterial infections that cause mastitis is milk. From a human milk sample, MALDI-ToF-MS successfully identified 56 (53.3%) streptococcal isolates at the species level (46). MALDI-ToF-MS was used to identify microbial diversity in 647 milk samples from women who had clinical symptoms of mastitis. In milk samples, the most common pathogens were *Staphylococcus epidermidis* (87.6%) and *Staphylococcus aureus* (22.1%), with Streptococcus (68.6%) being the second most common species (47). Colony culture of milk samples from cows with subclinical mastitis followed by MALDI-ToF-MS identified 106/120 (88.3%) at the genus and species level (score 2.0) and it was found to be more reliable than direct MALDI-ToF-MS after pre-incubation (48). Mass spectrometry alone is insufficientfor accurate and rapid pathogen detection,; a combined approach has proven to be more trustworthy while saving time. Consequently, three methods for identifying bacteria in milk samples from calved cows or with clinical mastitis were evaluated as agreement approaches: biochemical method, MALDI-ToF-MS, and 16S rRNA partial genomic sequence analysis. At the species level, *E. coli* and *S. aureus* were recognized, while others were identified at the genus level. Positive agreement was determined to be 94% among three approaches, and 95–98% between each pair of methods (49). With time, mass spectrometry has become more capable, and some laboratories are replacing biochemical approaches with MALDI-ToF-MS for the detection of microorganisms in milk samples. MALDI-ToF-MS was utilized by the researchers to match the bacterial isolates from the udder with other species in the database. Five hundred isolates were processed as bacterial colony material for this study, and 93.5% of them were recognized at the species level, while 6.5% were identified at the genus level. Those that were unable to be recognized at the species level were submitted to 16S rDNA sequencing. Streptococci, Staphylococci, Enterobacteriaceae, and Coryneforme are the most common bacteria (50). Wald et al. recently detected and distinguished *S. aureus* and coagulase negative Staphylococci in 200 milk samples from animals with clinical and subclinical mastitis, as well as cows with a somatic cell count of < 100,000 cells/ml (51). From subclinical mastitis milk samples, MLADI-ToF-MS found *S. argenteus* in seven isolates and *S. aureus* in eight (52). When MALDI-ToF-MS was compared to PCR-RFLP for detecting streptococci from milk samples, it was discovered that PCR-RFLP was more efficient and repeatable (53). Alnakip et al., on the other hand, recently compared MALDI-ToF-MS with 16S rRNA gene sequencing study to distinguish streptococci responsible for bovine mastitis. MALDI-ToF-MS was found to have a wide range of variability for detecting streptococcus at the species and sub-species level. It is clear that MALDI-ToF-MS is as powerful as 16S rRNA gene sequencing analysis, but it takes less time and is easier to do (54). Microbes can also be identified by mass spectrometry in other body fluids such as saliva, cerebrospinal fluids, and synovial fluids from humans and animals (55–57). It is past time to develop a combined MALDI-ToF-MS with instruments that will make it a standard and universal approach for the accurate detection of bacterial infections in all types of body fluids in clinical laboratories.

## 3. Role of proteomics to unravel bacterial pathogenesis

Proteomics tools are also contributing and improving with time in order to better understand the etiology of practically all bacterial illnesses. In fact, this technique has transformed this field by providing a straightforward and diverse way to learn about pathogenesis. Proteomics methods are commonly used to investigate virulence-related variables, oxidative stress, and the role of proteins in the host-pathogen interaction. Proteomics advancements have made it possible to investigate the hidden mechanisms of infections and identify the proteins involved. Pérez-Llarena and Bou (58), Katsafadou et al. (59), Yang et al. (60) have written some review studies in this area. We will highlight recent advancements in understanding bacterial pathogenesis in this portion of the review.

### 3.1. Gel based proteomics coupled with mass spectrometry

Bacterial proteins are widely known for their roles in virulence and other processes that aid bacteria in their pathogenicity (5). Proteomics' role in identifying virulent factors of key human pathogens such Mycobacterium TB, *Streptococcus pneumoniae*, and *Staphylococcus aureus* has been summarized (13, 61, 62). Gel-based proteomics, such as SDS-PAGE, 2-Dimensional gel electrophoresis (2-DE), and 2-Dimensional Differential Gel Electrophoresis (2-DDGE), are still popular methods for separating proteins before mass spectrometry analysis. They appear to be irreplaceable but have been improved with the addition of modern techniques. SDS-PAGE was used to segregate the whole cell proteome of *B. abortus* and *B. mellitensis*, which was then reacted with field sera from buffalo, cow, sheep, and goat. MALDI-ToF-MS was used to identify various important proteins such as heat shock proteins, binding proteins, hypothetical proteins, and enzymes. It was hypothesized that the antigens listed play a vital role in the pathogen's survival in the host cell environment (63). SDS-PAGE was used to isolate the phage protein PA-PP, which was then characterized using mass spectrometry (64). Li et al. created agarose native gel electrophoresis, which has been effectively applied to the characterization of antibodies in serum (65) as well as western blotting (66). SMA-PAGE, a technology combining styrene maleic acid lipid particles with this technique, was developed specifically for the separation of membrane proteins (67). Khan et al. employed 2-Dimensional gel electrophoresis to separate whole cell and membrane proteins extracted from *M. bovis* (68). The proteomes of high pathogenic (Staph 38) and less virulent (8325-4) strains of *Staph aureus*, which causes keratitis, were compared. Four binding proteins were discovered in less virulent strains using 2-DE and mass spectrometry, but many adhesions were found in staph 38, indicating its high virulence on the host cell surface (69). *Streptococcus suis* is a zoonotic bacterium that causes infections in pigs and humans, with symptoms such as meningitis, arthritis, and pneumonia. Two-Dimensional Differential Gel Electrophoresis (2-DDGE) was used to segregate the proteomes of two mutant strains, and differential proteins were discovered using label-free analysis. SBP2, or putative pilus protein, was discovered to be a novel pathogenic component of *S. suis* (serotype 2) that functions as a fibronectin and laminin adhesin (70). Nascimento Filho et al. summarized the role of proteomics methods in determining the virulence of the Leptospira pathogenic sp. that causes human leptospirosis (71). Many studies show that SDS-PAGE or 2-DE can be used to separate bacterial proteins and determine virulence factors. Proteome of *Compylobacter jejuni*, exo-proteome of *Clostridium difficle*, biofilm and adherence mechanism of *Vibrio parahaemolyticus*, identification of *C. jejuni* adhesion protein attached to the skin of slaughtered chicken, and fibronectin binding proteins in enteropathogenic *E. coli* O55:H7 are among the more recent studies (72–75). Pathogenesis is investigated by discovering the adhesion function of pathogenic proteins, as adhesion is the first step for bacteria to commence infection. Following predicted and applied proteomics, *M. bovis* nuclease demonstrated the ability to attach to macrophages and invade cells, as well as being cytotoxic to the host cells (76). *Chlamydia trachomatis* is a sexually transmitted disease that affects both men and women. Quantitative proteomics was used to detect its proteome during its replicative and infective stages in order to better understand its pathophysiology. Several proteins with metabolic activities were discovered using reverse phase two-dimensional UPLC followed by mass spectrometry (77). The persistence of *B. suis* in a host cell environment with reduced oxygen supply was investigated using the proteome and transcriptome. RegA was discovered to regress genes and proteins involved in metabolism and energy synthesis, particularly the Isocitrate Lyase gene (ICL). RegA's regression action inhibits pathogen metabolism, ensuring the infection's long-term survival in the host cell. ICL was discovered to be important in *B. suis* virulence and pathogenicity (78).

### 3.2. Other approaches of recent era

Quantitative proteomics is gaining popularity as a way to identify a group of proteins linked to a disease and achieve good results if the proteins aren't already separated on a gel. The proteomes of individuals with atopic dermatitis and healthy people were studied using the LC-MS-MS method. Some bacterial species, such as *Aeromonas hydrophila, Staphylococcus aureus*, and *Shewanella* sp., have been found to play a role in disease. Glyceraldehyde-3-phosphate, enolase, and chaperons like DnaK and HtpG were among the proteins found to be important in pathogenesis (79). Four acetyltransferases were discovered and described by mass spectrometry in *E. coli* (RimI, YiaC, YjaB, and PhnO). YiaC was a new protein discovered to play a role in flagellar motility and bacterial pathogenicity (80). By constructing the phosphoproteome followed by LC-MS-MS, the mechanism of protein phosphorylation related with *S. aureus* pathogenicity was elucidated. In comparison to previously reported mechanisms, Ser/Thr kinase signaling was found to be more efficient in virulence (81).

Another proteomic investigation analyzed the proteomes of ESBL and non-ESBL *Klabsiella pneumoniae* strains using nano LC-MS-MS. Stress proteins G and A, Lon proteases, and ElaB proteins were found to be shared between the two strains' proteomes. Furthermore, virulence-associated proteins such as lyase, oxidoreductase, catalase, and isochoristamase were discovered in ESBL *K. pneumoniae*, indicating that it is a more virulent strain (82). The quantity of pathogenic factors such as adenylate cyclase and O antigen was found to vary dramatically in the *Bordetella parapertussis* proteome using nano LC-MS-MS in limited iron circumstances. The research was expanded to look for proteins that were missing or thought to be pseudogenes in *Bordetella pertussis* in order to distinguish between the two species that cause whooping cough based on their virulence associated proteins (83).

The proteomes of *Salmonella typhimurium* wild type and fnr null mutant were characterized using label-free mass spectrometry. There were 153 significantly diverse proteins among the 1,798 discovered proteins, each responsible for a different metabolic activity. The fumarate nitrate reduction pathway in *Salmonella* regulates fis (DNA binding protein), a virulence related protein in *Salmonella typhimurium*, according to the findings (84). The extracellular and cell associated proteome profile of mutant and wild type strains of *Mycobacterium avium hominissuis* responsible for human infections was identified *via* label free analysis utilizing an LTQ Orbitrap Velos mass spectrometer. The lysX gene in mutant strains was discovered to be responsible for pathogen metabolic and virulence functions, as well as intracellular cell survival (85). Sputum and saliva from tuberculosis patients were exposed to quantitative proteomics utilizing the LTQ-Orbitrap technology in order to learn more about the processes that occur throughout the course of the disease. Proteins implicated in immunological regulation, complement activation, and inflammation were found in both samples. Uninfected people's samples contained a collection of proteins involved in pathogen protection and the innate immune response (86).

Two proteins (PRRC2C and RAB14) were identified using iTRAQ to have higher levels among 606 proteins, and three bacterial taxa (Streptococcus, Veillonella, and Haemophilus) were reported to have a tight relationship with chronic rhinosinusitis. Proteins linked with these bacteria were in short supply and served a variety of roles related to virulence and pathogenicity (61). In another investigation, iTRAQ was utilized to discover the *Lactobacillus acidophilus* differently expressed proteins at pH 7.4. A total of 207 proteins were found to be involved in carbohydrate and amino acid metabolism, as well as peptidoglycan production. At pH 7.5, adhesion-related proteins fmtB and PrtP were found to be increased, while anti-adhesion protein pyruate kinase was downregulated (87). In humans, *Acinetobacter baumannii* is known to cause nosocomial infections such as bacteremia, pneumonia, and meningitis, all of which have significant mortality and morbidity rates. Differential proteins were discovered using iTRAQ after infecting pigs' intestines with enterotoxigenic *E. coli* F4 (producing diarrhea in piglets) and pre-treating them with *Lactobacillus plantrum*. Cell division, differentiation, and cell cycle regulation were revealed to be connected with differentially expressed proteins between two bacterial species. The findings revealed ETEC intestinal epithelial cell processes and the protective effect of *L. plantrum* (88). Clearly, iTRAQ is the preferable technology for quantitative proteome analysis, as it provides a more trustworthy and comprehensive result. Another experiment measured the quantity of *Salmonella enteritidis* proteins in LB media supplemented with egg white and entire egg white. Protein abundance was observed to decrease as the amount of egg white was reduced using iTRAQ. Some virulence-related proteins were downregulated, while ABC transporters and co-factors were predominantly increased (89).

The abundance of ABC transporters and adhesion-related proteins in high pathogenic strains was discovered using LC-MS-MS and iTRAQ. It was also established that the sbp protein was implicated in the pathophysiology of the disease (90). iTRAQ coupled with 2D LC-MS-MS was used to investigate the proteome of *A. baimannii* standard strain and tigecycline-resistant strain. A total of 3,639 proteins were found, with 961 of them being differentially expressed. Differential proteins were linked to cellular component organization, stress responses, protein synthesis, protein degradation, and related functions, according to functional analysis. There were also some pathways linked to tigecycline resistance discovered (91).

However, additional quantitative proteome techniques have lately been applied. The abundance of outer membrane vesicles in coccoid was discovered utilizing a comparative proteome study of coccoid and spiral shaped *Helicobacter pylori* (gastric cancer) using the SILAC (stable isotopic labeling by amino acids in cell culture) proteome technique. Some proteins were discovered to be down regulated, including CagA, arginase RocF, and TNF-inducers (92). Another method for identifying isotope-labeled proteins is isotope dilution mass spectrometry. An isotope-labeled 15N-Cys C protein in *E. coli* was effectively discovered using this method (93). TMT (94, 95) is a method for quantifying proteins/peptides using tissue, serum, plasma, or other body fluid samples from the affected/diseased area. This has proven to be a reliable method for identifying proteins that are expressed at different phases of disease, allowing researchers to track disease pathophysiology and development (96). The aforementioned technique has recently been substituted by membrane coated nanosponges paired with quantitative proteomics technologies. This enhanced method proved to be quite beneficial in identifying bacterial toxins and/or pathogenic components (18). There are several mass spectrometry-based and isotopic labeled/label-free proteomics methods that have aided in the better understanding of the etiology of important bacterial diseases in humans and animals. Protein microarray is one of the advanced proteomics techniques of recent era. This includes antibody microarray in which proteins are labeled with captured antibodies, functional microarray uses purified proteins for various interactions and reverse-phase protein microarray finds its application in probing the target protein from cell lysates using antibodies (1).

### 3.3. Predictive proteomics

This method entails the use of bioinformatics tools to identify and test proteins based on their unique nature, structure, and functions. It is commonly used to anticipate the proteins produced by a certain bacterial pathogen and to identify the most important proteins linked to virulence. Gene ontology and enrichment analyses for function and pathway studies, as well as visualization tools to portray data in the form of graphs and charts, are the most crucial tools (97). This method was used to predict *Chlamydia pneumonia* nuclear targeting proteins that may play a role in lung cancer genesis (98). Computational biology and chemoinformatics were used to predict new therapeutic targets for *A. baumannii* (99). Baarda et al. compiled a list of tools that were useful in identifying vaccine candidates for *N. gonorrhoeae* (100). Using several bioinformatics tools available as web servers, secretory proteins of *M. bovis* were recently examined. Two proteins (MbovP274 and MbovP570) were chosen from the secretome data and experimentally confirmed to be immunogenic proteins (101). Many bacterial pathogens (*C. botulinum, C. defficile, Y. pseudotuberclosis, S. saprophyticus*, and *Legionella* sp.) have been studied *in silico* in order to find therapeutic targets and vaccine candidates (102–105). I-TASSER (https://seq2fun.dcmb.med.umich.edu//I-TASSER/) is an extensively used web server for the prediction of structure and function of the given protein (106) and Phyre2 (http://www.sbg.bio.ic.ac.uk/~phyre2/html/page.cgi?id=index) for protein modeling analysis (107). ConSurf web server (https://consurf.tau.ac.il/consurf_index.php) is usually used to identify the functional regions in protein (108). STRING (https://string-db.org/) is another online tool used for protein-protein interaction and functional predictions (109).

## 4. Identification of diagnostic markers

Proteins are a significant source of biomarkers and are used for illness diagnosis, prognosis, staging, and monitoring. Hormones, carbohydrate epitopes, enzymes, genetic alterations, and receptors are examples of biomarkers (110). Pathogen proteins have been shown to be responsible for virulence and infections, and hence can be used to find useful biomarkers for illness detection (111). The fact that they are important diagnostic indicators has piqued the interest of scientists all around the world in using proteomics technologies to uncover specific disease markers. Pasteurellosis and pneumonia in sheep have been proven to have biomarkers in the form of proteins and cytokines (112). Proteomics, both traditional and modern, is playing an increasingly important role in diagnostics, providing trustworthy and meaningful results. Since the last decade, mass spectrometry-based techniques have advanced significantly and are becoming increasingly useful in the search for promising diagnostic markers. Recent advances in quantitative proteomics, as well as increased accuracy, have paved the road for the discovery of effective diagnostic markers for a variety of disorders (113). The LC-MS-MS method is commonly used to diagnose diseases such as TB and periodontitis (114, 115). Table 1 depicts the many proteomics methodologies used to identify diagnostic markers for a certain disease.

**Table 1:** Diagnostic markers of various important bacterial diseases using proteomics tools.

**Pathogen**	**Disease**	**Methods**	**Diagnostic marker**	**References**
*Salmonella enterica*	Foodborne diseases	MALDI-ToF-MS	S8, L15, L17, L21, L25, S7, superoxide dismutase (SodA), peptidylprolyl cistrans isomerase C, Gns, YibT, YaiA, YciF	(116)
*Bacillus anthracis*	Anthrax	LC-MS, LTQ Orbitrap	SASP-gamma, 30S ribosomal protein S10, putative lipoprotein, and 60 kDa chaperonin proteins	(117)
*Chlamydia trachomatis*	Sexually transmitted infections	SDS-PAGE, Western blot	TroA, HtrA	(118)
*Brucella canis*	Canine brucellosis	Recombinant proteins, iELISA	PdhB, Tuf proteins	(119)
*Leptospira* species	Leptospirosis	Predictive proteomics	LipL32 protein	(120)
*Brucella melitensis*	Brucellosis	Recombinant protein, western blot	virB10 protein of T4SS	(121)
*Staphylococcus aureus*	Bone and joint infections	MALDI-TOF	Delta-toxin	(122)
*Nocardia farcinica* IFM 10152	Nocardiosis	Predictive proteomics, MALDI-TOF-MS western blot	NFA_45140, NFA_55680, NFA_48660, NFA_49580, NFA_15900	(123)
*Salmonella enteritidis*	Salmonellosis	LC-MS-MS	AHSG, VNN1	(124)
*Campylobacter Jejuni*	Gastroenteritis	Protein microarray, immunoblotting	Cj0144, Cj0262c, Cj1621, GreA, and PrfA, CjaA	(125)
*Escherichia coli*	Urinary tract infections	iTRAQ	RbsB, YoeA, BamA, GroEL	(126)
*Mycoplasma bovis*	Calf pneumonia	2-DE, Western blot, MALDI-TOF-MS	P579	(68)
*Mycoplasma agalactiae*	Keratoconjunctivitis, mastitis, still birth, vulvovaginitis	Predictive proteomics, western blotting	MAG_1560, MAG_6130, P40	(127)
*Mycobacterium tuberculosis*	Tuberculosis	2-DE, LC-MS-MS	mmsA, pntAa	(128)
*Mycobacterium bovis*	Bovine tuberculosis	Predictive proteomics	Mb0854c, Mb2898	(129)
*Pasteurella multocida*	Fowl cholera	Recombinant protein purification, iELISA	rOmpH protein	(130)
*Helicobacter pylori*	Gastritis, peptic ulcer	Recombinant protein purification, western blot, sandwich ELISA	FliD Protein	(131)
*Mycoplasma pneumonia*	Pneumonia	Immunoblot, ELISA	heptapeptide 1 & heptapeptide 2	(132)
*Yersinia pestis*	Plague	Western Blot, immunochromatography	F1 antigen	(133)
*Mycobacterium leprae*	Leprosy	Recombinant protein, western blot, ELISA	rMLP15 antigen	(127)
*Borrelia burgdorferi*	Lyme disease	Mass spectrometry, microarray	DbpA, Fla, VIsE, p83/100, BB_G31, BB_J48	(134)
*Mycoplasma hyopneumoniae*	Swine pneumonia	Recombinant protein, western blot, ELISA	Mhp366 protein	(135)
*Mycoplasma bovis*	Calf pneumonia	Predictive proteomics, western blot, adhesion assay	NADH oxidase as adhesion and NADH oxidizing and O_2_ reducing enzyme	(136)
*Mycoplasma bovis*	Calf pneumonia	Predictive proteomics, binding assay	P27 as fibronectin binding adhesion	(137)
*Mycoplasma bovis*	Calf pneumonia	2DE, MALDI-ToF MS, LC-MS/MS	MbovP730 as DIVA antigen	(138)
*Mycoplamsa hyorhinis*	Swine synositis, meningitis, lameness	Colony blot, binding assay	GAPDH moonlights as adhesin and ECM degradation protein	(139)
*Mycobacterium tuberculosis*	Tuberculosis	2DE, MALDI-ToF-MS	miR-625-3p, mannose-binding lectin 2, inter-α-trypsin inhibitor H4 as combined diagnostic biomarker	(140)
*Coxiella burnetti*	Q fever	Predictive proteomics	CBU1910 (Com1), CBU1718 (GroEL), CBU0236 (Tuf-2), CBU0092 (YbgF), and CBU0612 (OmpH)	(141)
*Bartonella bacilliformis*	Carrion's disease	Predictive proteomics	Flagellar biosynthetic protein, heme exporter protein C, Cytochrome c-type biogenesis protein, Hemin ABC transporter, phosphatidate cytidylyltransferase	(142)

The improved mass spectrometry approach for the absolute detection of biomarkers from Salmonella serotypes was introduced by Fukuyama and colleagues (116). In a cohort research, quantitative proteomics was used to uncover distinct biomarkers in the plasma of individuals with active tuberculosis. Five proteins, CFHR5, LRG1, CRP, LBP, and SAA1, have been discovered to clearly distinguish tuberculosis patients from those with other respiratory illnesses (143). The protein microarray technique was utilized to identify diagnostic indicators for *Salmonella typhi*, and it was found to be highly repeatable (144). In another cohort investigation, the whole proteome microarray approach was employed to identify protein biomarkers from *Chlamydia trachomatis*. A total of 121 antigens were discovered, 18 of which might be used as diagnostic markers. Furthermore, the antigens CT 858, CT 813, and CT 142 were thought to represent possible disease markers in the future (145). Considering the findings of recent studies, it is clear that proteomics technologies are playing an important role in illness diagnoses.

## 5. Proteomics and secretome

The secretome is a collection of proteins that are either released in soluble form or overlapped by vesicles from bacterial cells. These secreted proteins play a critical role in bacterial virulence, and their characterization has become increasingly relevant in the quest better understanding bacterial virulence. Proteomics technologies have proven to be extremely useful in this procedure, from secretome extraction to characterization (146). Bioinformatics and other proteomics methods, including as mass spectrometry and immunoproteomics, have contributed in the discovery of antigenic secreted proteins and vaccine candidates (147). Table 2 illustrates the secretomes of pathogenic bacteria and the proteins that have been identified as virulent factors, protective antigens, or vaccine candidates.

**Table 2:** Secreted proteins from important bacterial pathogens using proteomics tools.

**Pathogen**	**Disease**	**Methods**	**Outcome**	**References**
*Mycoplasma capricolum subsp. Capricolum*	Caprine arthritis, mastitis, respiratory diseases	2-DE, MALDI-TOF	Acid phosphatase, hemolysin, gelatinase as virulent factors	(148)
*Salmonella enterica serovar Typhimurium*	Typhoid fever	LC-MS-MS, western blot	SopF effector	(149)
*Bordetella pertussis*	Whooping cough	Predictive proteomics, LC-MS-MS	PtxA and CyaA, TcfA, FhaL and FhaS, BP1251	(150)
*Streptococcus pyogenes*	Pharyngitis, necrotizing fasciitis	label-free LC–MS-MS	HtpA as virulence associated effector protein	(151)
*E. coli* (AIEC), (ETEC)	Intestinal and extra-intestinal diseases in humans	1D SDS-PAGE, LC-ESI-MS-MS	LF82_130, adhE, ykgD,rclR, ycdB, fhuA, fabF, traV, ETEC_4010, ETEC_2119, ETEC_2033. ETEC_0806, gapC, yfdQ, glyA, adhE, fabF identified as vaccine candidates	(152)
*Streptococcus pneumonia*	Pneumonia, bacteremia, meningitis	1-DE, LC-MS-MS	Gsp-781, Sphtra, NagA, PhtD, ZmpB, Eno as immunogenic proteins	(153)
*Propionibacterium acnes*	Acne vulgaris	Mass spectrometry	PPA1939 as vaccine candidate	(154)
*Helicobacter pylori*	Peptic ulcer	Predictive proteomics	vacA, babA, sabA, fecA and omp16 as vaccine candidates	(155)
*Mycoplasma bovis*	Calf pneumonia	Predictive proteomics, western blot, binding assay	MbovP280 a novel secreted protein inducing apoptosis *via* C-C domain and ligand CRYAB	(156)
*Mycoplasma bovis*	Calf pneumonia	Predictive proteomics, label free quantitative proteomics	MbovP0145 as potential diagnostic marker	(157)
*Mycoplasma bovis*	Calf pneumonia	Predictive proteomics, western blot	MbovP0145 induces IL-8 expression through MAPK pathway	(158)
*Campylobacter jejuni*	Gastroenteritis	SILAC, label free LC-MS-MS, immunoblot	CJM1_0791 and CJM1_0395 virulent proteins	(159)
*Mycobacterium bovis* 04-303	Tuberculosis	LC-MS-MS	EsxA and EsxB	(160)
*Mannheimia haemolytica*	Bovine respiratory disease	LC–MS-MS, predictive proteomics	Several Immunogenic secreted proteins	(161)
*Mycoplasma hyopneumoniae* and *Mycoplasma flocculare*	Porcine enzootic pneumonia	LC-MS-MS	15 Proteins in *M. hyponeumoniae* and four in *M. flocculare* as potential virulent factors	(162)
*Streptococcus pneumonia*	Pneumonia, septicemia	MALDI-TOF-TOF, immunoblot	tatD- endodeoxyribonuclease as virulent factor	(163)
*Mycoplasma bovis*	Calf pneumonia	MALDI-TOF-MS	rMbovP581 as immunogenic protein	(8)
*Brucella* rough mutants	Macrophage death	SDS-PAGE, LC-MS-MS	BAB1_1579, BAB1_1185 as cytotoxic proteins	(164)
*Francisella tularensis*	Tularemia	Mass spectrometry, western blot	OpiA, OpiB, PdpC, and PdpD as virulence effector proteins	(165)
*Mycobacterium tuberculosis*	Tuberculosis	Recombinant proteins, SDS-PAGE	CFP-10, ESAT-6 as diagnostic markers	(166)
*Rickettsia*	Rickettsioses	Literature review, predictive proteomics	Sca4, RickA, RalF, TlyC, PLD, Pat1, Pat2 for better understanding of secretion system and virulence	(167)
*Shigella flexneri*	Bacterial dysenteries, shigellosis	Mass spectrometry, Immunoblot	Orf13 and Orf131a as virulence effector proteins	(168)
*Bacteroides fragilis*	Bowel disease, colon cancer	HPLC-MS-MS, proteogenomics	Metabolic activity of pathogenic strain EVs indicate more pathogenic potential as compared to non-pathogenic	(169)
*Vibrio cholerae*	Cholera	Mass spectrometry, western blot	TseH, TsiH	(170)
*Staphylococcus aureus*	Skin infections	LC-MS-MS, immunoblot	cytolysins	(171)
*Streptococcus suis*	Swine Septicemia, meningitis, arthritis, endocarditis	LC-MS-MS, immunoblot	SSU0020, SSU0934, and SSU0215 as vaccine candidates	(172)
*Mycoplasma bovis*	Calf pneumonia	Predictive proteomics	14 putative secreted proteins associated with virulence	(173)
*Bacillus anthracis*	Anthrax, bioterrorism agent	Predictive proteomics	Anthrolysin, BsIA, PA domain 4, LF domain 1, EF as candidates for chimeric vaccine	(174)
*Leptospira interrogans*	Leptospirosis	Mass spectrometry- LTQ-Orbitrap	Secreted protease unable to degrade human plasmin & ECM	(175)

**Figure 2:**
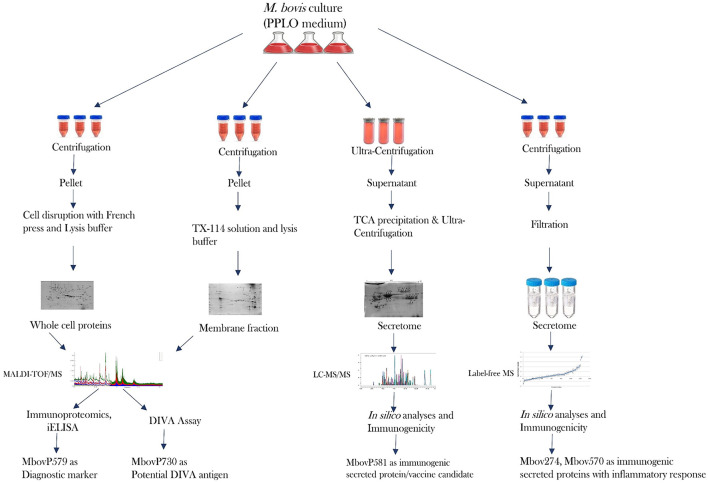
Recent progress in the proteome and secretome of *M. bovis* and its outcome.

It is clear that proteomics methods are extremely useful for studying and characterizing the bacterial secretome in depth. The limits for identifying really secreted proteins are a crucial point to make here. Bioinformatics alone is insufficient to achieve this goal (176). A highly advanced proteomics technique is required to identify proteins that were originally produced by specific bacteria. Many *in silico* studies have been carried out, however there is still some confusion concerning protein secretion and the mechanisms involved, such as classical and non-classical secretion (167, 173, 177–179). Another area of debate that gets mixed up with the bacterial secretome is cell lysis and secretion of non-classical proteins (180, 181). Visual proteomics is a viable approach for identifying bacterial extracellular vesicles in a sample, but due to their small size, released soluble proteins are not visible by SEM or TEM. Because proteins are only projected to be secreted *via* multiple secretion pathways, there is a pressing need to develop a better proteomics tool. This allows one to identify truely secreted proteins as well as their secretion pathways, removing the ambiguities associated with prediction tools. A review gives light on the challenges of extracting and characterizing the bacterial secretome, particularly in the case of *Mycoplasma* sp. (176). Because serum in growth media interferes with secreted proteins, many people utilize media with lower serum concentrations (8, 162, 182). It is now recommended that serum-free media be used for *Mycoplasma* sp. culture in order to discover proteins of interest without disrupting serum proteins. If this can be accomplished without affecting growth or cell lysis, it will be a significant contribution to the field of proteomics for *Mycoplasma* sp. In terms of proteomics, *Mycoplasma bovis* has been a widely investigated bacterium in recent years. Using entire cell proteins, membrane proteins, and secreted proteins, effective research has recently been published in order to uncover diagnostic markers and vaccine candidates (8, 68, 101, 173). Profiling core secretome proteins among different strains of pathogenic bacteria might be significant to future studies as supported by the recent core genome studies (183). Figure 2 depicts the advancement of the proteome of *Mycoplasma bovis* in a schematic manner, which could be extremely useful in filling gaps in proteomics study of other significant *Mycoplasma* sp. such as *M. hyorhinis, M. hyopneumoniae, M. agalactiae*, and *M. mycoides* sub sp *mycoides*.

## 6. Concluding remarks

Proteomics has played a vital role in identifying and distinguishing bacterial infections, as well as understanding and diagnosing their pathophysiology. Using a combination of methods, researchers were able to more effectively detect infections as well as identify and characterize the proteins involved in pathogenicity. Proteomics enabled to detect the secretome of bacterial pathogens, in addition to entire cell and membrane proteins, and gave a new platform for the field of preventive medicine. In order to confirm and describe the secretory nature of proteins implicated in bacterial pathogenicity, more progress must be made.

## Author contributions

MZ wrote the manuscript. YY, JW, and AS collected the literature. MF set the tables. MQ designed the figures. ZF and GS revised the manuscript. YW and QX organized the contents and revised the manuscript. All authors contributed to the article and approved the submitted version.
